# Development of Time-Resolved Fluoroimmunoassay for Detection of Cylindrospermopsin Using Its Novel Monoclonal Antibodies

**DOI:** 10.3390/toxins10070255

**Published:** 2018-06-21

**Authors:** Lamei Lei, Liang Peng, Yang Yang, Bo-ping Han

**Affiliations:** Department of Ecology and Institute of Hydrobiology, Jinan University, Guangzhou 510632, China; tleilam@jnu.edu.cn (L.L.); tpengliang@jnu.edu.cn (L.P.); yangy@jnu.edu.cn (Y.Y.)

**Keywords:** cylindrospermopsin, monoclonal antibody, time-resolved fluoroimmunoassay, method validation, detection

## Abstract

Cylindrospermopsin (CYN) is a cyanotoxin that is of particular concern for its potential toxicity to human and animal health and ecological consequences due to contamination of drinking water. The increasing emergence of CYN around the world has led to urgent development of rapid and high-throughput methods for its detection in water. In this study, a highly sensitive monoclonal antibody N8 was produced and characterized for CYN detection through the development of a direct competitive time-resolved fluorescence immunoassay (TRFIA). The newly developed TRFIA exhibited a typical sigmoidal response for CYN at concentrations of 0.01–100 ng mL^−1^, with a wide quantitative range between 0.1 and 50 ng mL^−1^. The detection limit of the method was calculated to be 0.02 ng mL^−1^, which is well below the guideline value of 1 μg L^−1^ and is sensitive enough to provide an early warning of the occurrence of CYN-producing cyanobacterial blooms. The newly developed TRFIA also displayed good precision and accuracy, as evidenced by low coefficients of variation (4.1–6.5%). Recoveries ranging from 92.6% to 108.8% were observed upon the analysis of CYN-spiked water samples. Moreover, comparison of the TRIFA with an ELISA kit through testing 76 water samples and 15 *Cylindrospermopsis* cultures yielded a correlation *r*^2^ value of 0.963, implying that the novel immunoassay was reliable for the detection of CYN in water and algal samples.

## 1. Introduction

Cyanobacterial blooms occur frequently in eutrophic freshwater lakes, reservoirs and rivers throughout the world. Many cyanobacterial species are capable of producing cyanotoxins that pose a significant threat to both water quality and human health [1]. Cyanotoxins are a variety of secondary metabolites that include microcystins (MCs), nodularin, cylindrospermopsin (CYN), anatoxin-a, and saxitoxins [2]. Cylindrospermopsin is becoming one of the most commonly studied cyanotoxins because of its toxicity and increasing presence in different environments [3,4]. Several cyanobacterial species, such as *Cylindrospermopsis raciborskii*, several *Aphanizomenon* species and *Raphidiopsis curvata*, have been reported to be potent CYN-producers [5,6,7]. Cylindrospermopsin-producing cyanobacteria has been detected in Australia and New Zealand, Asia, South and North America, West Africa, and Europe [4,8].

Cylindrospermopsin is a guanidine alkaloid that has an LD_50_ of 2.1 mg kg^−1^ over 24 h after intraperitoneal administration to mice [5]. Exposure to CYN rapidly increased the production of reactive oxygen species (ROS) and may result in serious cytotoxic and genotoxic effects [3,9,10]. Oxidative stress is one of the key mechanisms involved in CYN toxicity [9,10]. Moreover, CYN was found to suppress lymphocytes proliferation and could be classified as a potential immunotoxicant [9,11,12]. The alkaloid is probably more hazardous to human and animal health than microcystins (MCs) because of its cell transforming potential [13]. When compared to other cyanotoxins, CYN is more stable under a wide range of pH and temperatures, and may present significant consequences for aquatic environments [14]. Thus, qualitative and quantitative analytical tools need to be developed for long-term monitoring of CYN in freshwater to minimize its risks to water quality and human health.

Common approaches to the detection of cyanobacteria and their toxins in the environment are currently chemical-, biochemical-, or molecular-based methods [3,15]. Specifically, analytical methods include high-performance liquid chromatography-photo-diode array (HPLC-PDA), liquid chromatography-mass spectrometry (LC-MS/MS), enzyme-linked immunosorbent assay (ELISA), and conventional or real-time PCR assays [16,17,18,19], with LC-MS/MS the most commonly used [16,20,21]. However, LC-MS/MS relies on specialized and expensive equipment. The development of immunological approaches has yielded more sensitive, rapid and high-throughput tools for the detection and quantification of cyanotoxins in all kinds of water and cultured samples [14]. To establish immunoassays, monoclonal or polyclonal antibodies are required to be raised against CYN. Two commercial ELISA kits based on rabbit anti-CYN polyclonal antibodies are currently available with low detection limits of 0.1 ng/mL (Beacon Analytical Systems Inc., Saco, ME, USA) and 0.05 ng/mL (Abraxis LLC, Warminster, PA, USA), respectively [15]. Both kits have been used to determine CYN in raw water and cyanobacterial extracts [19,20,22,23,24,25,26,27]. Elliott et al. [28] published the first detailed report on the production of polyclonal and monoclonal antibodies to CYN. These antibodies were employed in competitive indirect ELISA, an optical biosensing technique of surface-plasmon resonance (SPR), the Luminex method and the MBio Biosensor [28,29,30]. However, more antibodies with high specificity to CYN are required for further development to increase the sensitivity and applicability of the different immunoassays.

Time-resolved fluorescence immunoassay (TRFIA) uses lanthanide chelates and has been widely used for clinical screening and diagnostics. Lanthanide chelates have unique luminescent properties, such as a long Stokes’ shift and exceptional decay times, which allow for efficient temporal discrimination of background interferences in the assays [31]. The technique TRFIA is characterized by a long storage time, high sensitivity and specificity, good repeatability and wide detection range with no radioactive contamination [32,33]. In this study, specific monoclonal antibodies to CYN were produced and used to improve on the TRFIA method to develop a sensitive immunological technique for quantification of CYN.

## 2. Results and Discussion

**Figure 1:**
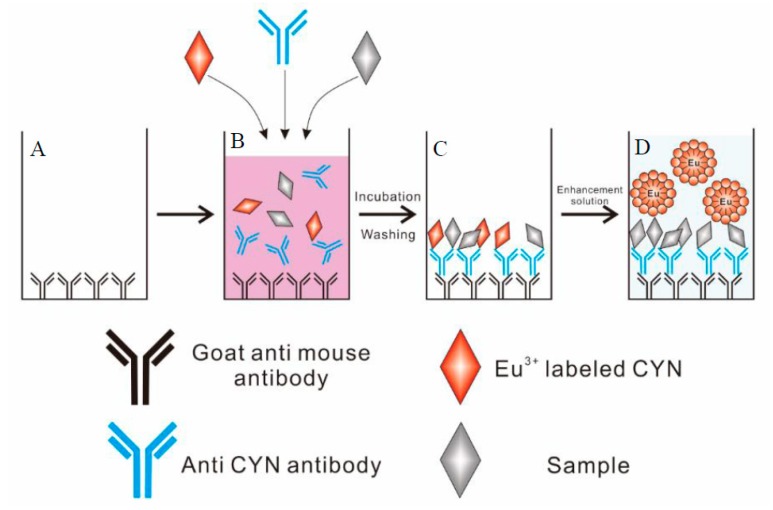
Example of the newly developed TRFIA employing a europium chelate label. (**A**) adsorption of goat anti-mouse antibody; (**B**) a direct competitive reaction; (**C**) formation of CYN/antibody complex; (**D**) measurement of fluorescence intensity.

Cylindrospermopsin is becoming one of the most commonly studied cyanotoxins because of its wide distribution and multiorgan toxicity, and its ecological role and triggering environmental factors have not fully been understood; however, there are relatively few methods available for its analysis [34,35,36]. In this study, we developed a novel direct competitive TRFIA technique to measure CYN at trace levels (Figure 1). Our TRFIA provides a fast and highly sensitive screening method and may act as an early warning detection tool for CYN monitoring.

### 2.1. CYN Conjugate Preparation

Like other cyanotoxins, CYN is a low-molecular-weight nonimmunogenic toxin. To become immunogenic, these toxins need be conjugated to carrier proteins by means of different chemical approaches [28,37]. The Mannich reaction, a conjugation between one active hydrogen and primary amines in the presence of formaldehyde, has been used to link another alkaloid toxin-saxitoxin (STX) to BSA, while polyclonal antibody R895 was generated using the conjugate as an immunogen [38]. Among five chemical approaches to synthesizing CYN immunogens, the modified Mannich reaction was considered the most effective method for antibody production [28]. Although many carrier proteins have been used in the coupling of cyanotoxins, BSA and KLH are the most common ones. In the present study, we used BSA and KLH to synthesize CYN conjugates via the Mannich reaction and then obtained an immunogen (KLH-CYN) and coating antigen (BSA-CYN).

### 2.2. Monoclonal Antibody Production

Hybridized cells were plated on six 96-well plates and screened by ELISA for monoclonal antibody production. The first screening with BSA-CYN yielded 64 antibody-producing clones, while the second screening yielded 22. Next, 22 cell lines were re-cloned twice, after which nine clones remained. The subclasses of these nine positive clones determined by the Pierce^®^ Rapid Isotyping Kit were N1 (IgG1 λ), N2 (IgG1κ), N3 (IgG2a λ), N4 (IgG1 λ), N5 (IgG1κ), N6 (IgG2a λ), N8 (IgG1 λ), N10 (IgG1 λ) and N16 (IgG1 λ).

**Figure 2:**
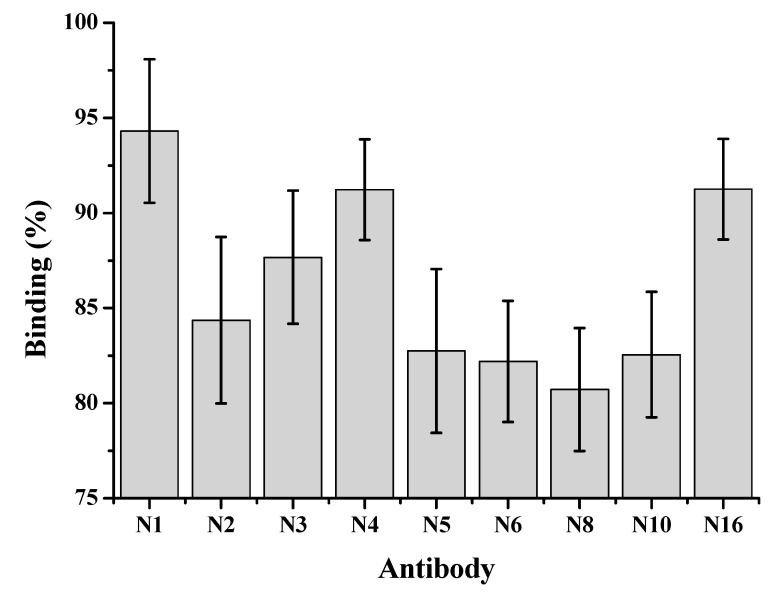
Percentage binding of nine monoclonal antibodies to BSA-CYN in the presence of 0.1 ng mL^−1^ CYN.

The binding capacity of different MAbs to CYN was examined by a direct competitive TRFIA: 0.1 ng mL^−1^ CYN standard, an appropriate dilution of Eu^3+^-labeled BSA-CYN, and addition of each MAb to the plate coating with goat anti-mouse IgG. After incubation, washing and fluorescence measurement was determined as follows:

binding % = (*F*/*F*_0_) × 100

where *F* corresponds to the fluorescence value of wells in the presence of 0.1 ng mL^−1^ CYN, and *F*_0_ is the fluorescence in the absence of CYN. The binding of all nine MAbs to BSA-CYN can be inhibited by low concentration of free CYN, but the inhibition varied significantly and N8 had higher affinity to CYN than the other eight antibodies (Figure 2).

To the best of our knowledge, this was the second detailed investigation of CYN antibody production after Elliott et al. [28]. Furthermore, rabbit anti-CYN polyclonal antibodies have been employed in the immunoassays according to the instructions of commercial ELISA kits, but no information regarding antibody production is available. Suitable antibodies are essential to the establishment of immunoassays. In this regard, MCs have received a great deal of attention, and several types of antibodies including conventional polyclonal or monoclonal antibodies and novel recombinant antibodies have been developed [37,39,40,41]. Moreover, antibodies have been raised specifically against some MC variants [40,42,43]. Weller [44] pointed out that the production of high quality antibodies would be highly desirable for all other cyanotoxins, a situation that caused delays to the development of multianalyte immunoassays and biosensors. Apparently a gap exists in both antibody production to CYN and its immunological techniques. Therefore, our novel monoclonal antibody N8 has wide application prospects.

### 2.3. Establishment of Standard Curves

**Figure 3:**
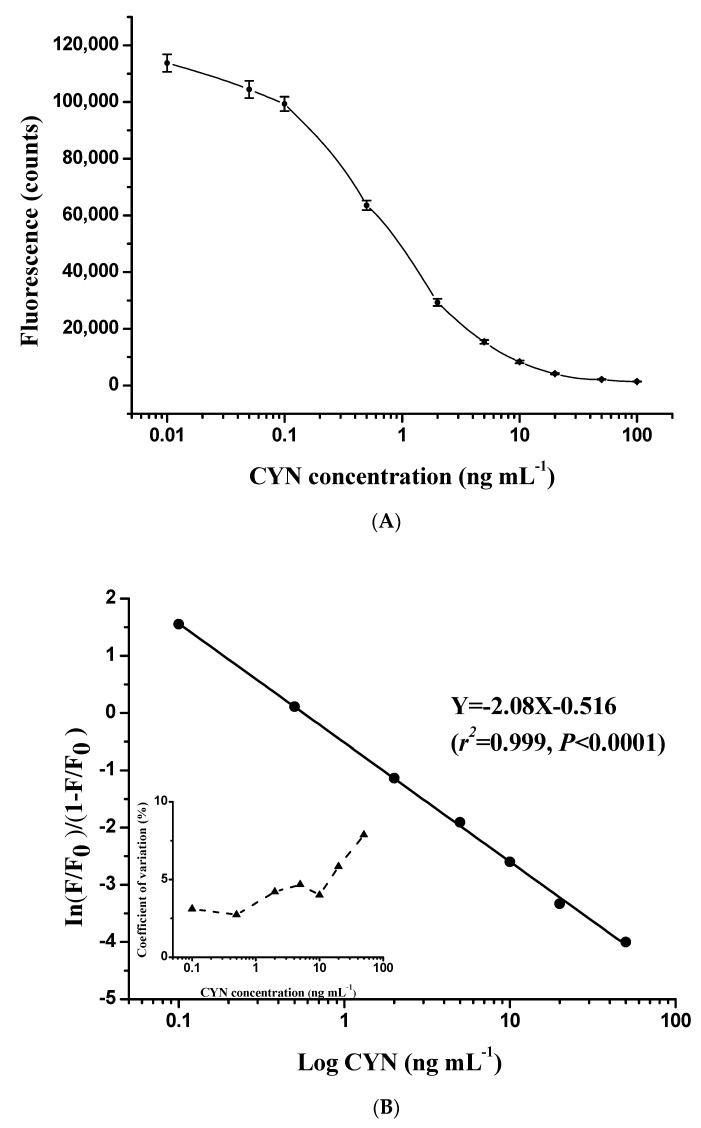
Typical standard curve showing the normalized fluorescence signal as a function of CYN concentration in the range of 0 to 100 ng mL^−1^ (**A**). The corresponding logit-log linear calibration curve and intra-assay precision profile (each point was based on 10 replicates) are shown in (**B**).

The direct competitive TRFIA curve established with antibody N8 showed a typical sigmoidal response for CYN at concentrations of 0.01–100 ng mL^−1^ (Figure 3A). In addition, the fluorescence signal was evaluated following our immunoassay design with a serial dilution of standards obtained from 10 separate assays. The coefficient of variation of each standard was less than 10%, indicating high reproducibility of the TRFIA curve. The logit-log method was used for curve fitting, and the best-fit calibration fell into the concentration range of 0.1–50 ng mL^−1^ (Figure 3B). Concentration of CYN in the sample is quantitatively determined by the logit-log model:

$$\mathrm{In}\left\{\frac{\frac{F}{F_{0}}}{1-\frac{F}{F_{0}}}\right\}=-2.08\times \mathrm{LogX}-0.516$$

where *F* is the fluorescence value of wells with the unknown sample, *F*_0_ is the fluorescence value of zero concentration, and X is the CYN concentration of the unknown sample.

In this way, a TRFIA was established to detect CYN in environmental samples. This immunoassay has been applied previously to detect microcystins and nodularin, and some advantages such as a wide detection range and high sensitivity have been demonstrated [33,45,46]. In our study, the TRFIA also exhibited a wide quantitative range between 0.1 and 50 ng mL^−1^ and maintained good reliability. We did not test the cross-reactivity of N8 to other CYN variants because no commercial standard was available. According to a previous study [28], polyclonal and monoclonal antibodies raised against CYN showed relatively low cross-reactivities with deoxy-CYN. However, the CYN ELISA kit from Abraxis LLC appeared to provide good quantification for the assessment of CYN and its variants [27]. The difference in performance may be attributed to antibody specificity, and some antibodies with broad specificity are capable of recognizing several cyanotoxins with similar structures [37].

### 2.4. Assay Validation

#### 2.4.1. Analytical Sensitivity of the Present Method

Under the optimized conditions, the detection limit of the assay was 0.02 ng mL^−1^ CYN. Sensitivity of the newly developed TRFIA method is similar to that of the monoclonal antibody-based ELISA of Elliott et al. [28] and the Abraxis ELISA kit, but higher than that of the monoclonal antibody-based SPR of Elliott et al. [28] and the MBio assay of McNamee et al. [30]. Both TRFIA and commercial ELISA kits are capable of detecting CYN within the guideline value of 1 μg L^−1^ proposed by Humpage and Falconer [35].

#### 2.4.2. Precision and Accuracy of the Present Method

Table 1 shows the precision of the developed TRFIA for the quantitative detection of CYN. Coefficients of variation for both intra- and inter-assays ranged from 4.1% to 6.5%. Precision of the present assay was excellent, and none of the coefficients of variation were significant (≥10%). The general analytical recovery of the assay was in the range of 90–110%, indicating the high accuracy of the measurements.

#### 2.4.3. Recovery of the Developed Method

The recoveries of CYN-spiked water samples ranged from 92.6% to 108.8%, with coefficients of variation <16.38% (Table 2). The results showed that our TRFIA was not affected by the matrix of the natural environment when detecting CYN in water samples.

#### 2.4.4. Dilution Linearity for the Present Method

Table 3 shows the dilution linearity results of the assay when we used positive samples serially diluted with our assay buffer. The expected values were derived from the initial value of potency in the undiluted samples. We found that the expected values were almost identical to the measured values, as evidenced by the high recoveries (94.5–108.7%). These results confirmed that linearity was good over a wide range of dilution and detection would be unaffected if the sample was diluted with assay buffer. Therefore, the TRFIA method provides flexibility to assay samples with distinct levels of CYN.

### 2.5. Comparison of Assay Results and Performance of the Developed TRFIA and ELISA

**Figure 4:**
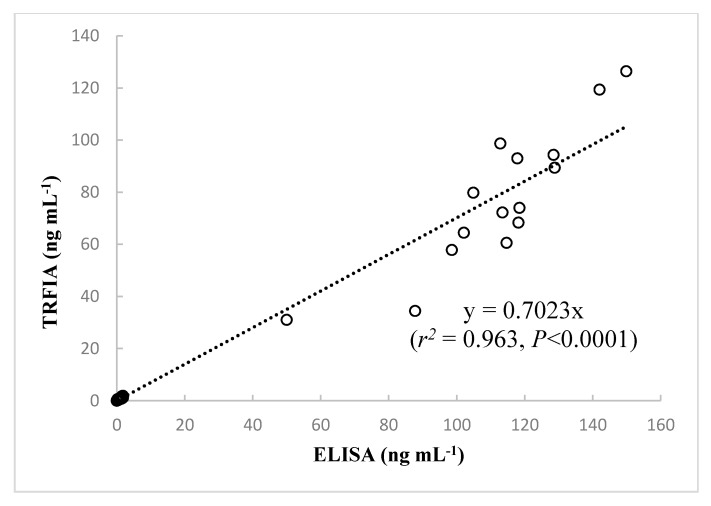
Correlations analysis between CYN concentrations measured by the newly developed TRFIA and by ELISA in 91 samples. Data represent the means of three determinations.

The assay performance of the developed TRFIA and the performance data provided in the instruction manuals of the commercial ELISA kits are compared in Table 4. It should be noted that the TRFIA method was faster, more precise, and had a wider detection range than the ELISA kits. To investigate reliability of the developed TRFIA for measurement of CYN in different samples, a total of 91 samples (76 water samples collected from Guangdong reservoirs in South China and 15 *Cylindrospermopsis* cultures) were assayed by TRFIA and ELISA. There were 84 positive samples identified by TRFIA and 83 by ELISA, indicating good agreement between the two methods. The results of our method are compared to those of the Beacon ELISA kit in Figure 4. The estimated contents obtained from the present TRFIA method and the Beacon ELISA kit showed very high correlation (*r*^2^ = 0.963, *p* < 0.0001, *n* = 91); thus, the novel immunoassay developed by our group can be considered a useful tool for detection of CYN in water and algal samples.

We can infer from the large positive correlation coefficient that examination of CYN samples by ELISA resulted in slightly higher reported concentrations than TRFIA. Previous studies suggest that ELISA may overestimate the CYN concentration [20,22,47]. Moreover, Metcalf et al. [27] found that the non-cyanotoxin-producing green alga, *Chlorella* sp., also gave positive responses in ELISA. The qualitative difference might result from undesired cross-reactivity of antibodies used in ELISA [47]. The present TRFIA was developed using N8 monoclonal antibody; however, commercial ELISA kits typically use rabbit anti-CYN polyclonal antibodies. For the polyclonal sera raised against CYN-OVA, a high level of non-specific background response was observed when evaluated by ELISA [28]. The high specificity of the monoclonal antibody may reduce the probability of cross reactivity and non-specific binding [48]. Future work is needed to evaluate the cross-reactivity of N8 monoclonal antibody to CYN analogues namely deoxy-CYN and 7-epi-CYN. Additionally, LC-MS/MS has been developed as the ideal confirmation technique for trace CYN in environmental samples, and comparison of the present TRFIA method with LC-MS/MS is highly desirable for further confirming the accuracy and reliability of the TRFIA.

## 3. Materials and Method

### 3.1. Chemicals and Solutions

Pure CYN (purity > 95%) was obtained from Enzo Life Sciences (Farmingdale, NY, USA). Keyhole limpet hemocyanin (KLH), bovine serum albumin (BSA), 2-morpholinoethanesulfonic acid (MES), Jeffamine, EDC, N-hydroxysuccinimide (NHS) and other reagents were purchased from Sigma–Aldrich (St. Louis, MO, USA). Goat anti-Mouse IgG secondary antibody was acquired from Biodesign International (Saco, ME, USA) and Eu^3+^ labeled kits were obtained from PerkinElmer (Turku, Finland).

The MES buffer consisted of 50 mM 2-morpholinoethanesulfonic acid with 500 mM NaCl (pH 5), while the coating buffer was 50 mM Na_2_CO_3_-NaHCO_3_ buffer (pH 9.6) and the blocking solution was 50 mM Na_2_CO_3_-NaHCO_3_ buffer (pH 9.6) containing 1% BSA. The labeling buffer was 50 mM Na_2_CO_3_-NaHCO_3_ (pH 8.5) with 155 mM NaCl, elution buffer was 50 mM Tris-HCl (pH 7.4) with 0.2% BSA and 0.9% NaCl, standard buffer was 50 mM Tris-HCl (pH 7.8) containing 0.1% NaN_3_ and 0.2% BSA, and assay buffer was 50 mM Tris-HCl (pH 7.8) with 0.02% BSA, 0.05% Tween-20 and 0.05% NaN_3_. The enhancement solution was 100 mM acetate-phthalate buffer (pH 3.2) containing 15 μM β-naphthoyltrifluoroacetate, 50 μM tri-n-octylphosphine oxide and 0.1% triton X-100. The washing buffer was 25 mM Tris-HCl (pH 7.8) with 0.9% NaCl and 0.06% Tween-20.

### 3.2. Preparation of Protein Conjugates KLH-CYN and BSA-CYN

The KLH-CYN protein conjugate was prepared using a modification of the Mannich reaction as described by Elliott et al. [28]. Cylindrospermopsin (250 μg) was added to KLH (1.5 mg) dissolved in 200 μL of phosphate buffer. Formaldehyde (6 μL) was then added, after which the mixture was stirred in the dark at room temperature for 50 h. The conjugate was subsequently purified by dialysis in 0.15 M saline solution.

The BSA-CYN protein conjugate was prepared using a modification of the Mannich reaction described by Compbell et al. [38]. An aliquot (250 μL) of EDC (20 mg) and NHS (8 mg) dissolved in MES buffer was added to the BSA (10 mg dissolved in MES buffer) and mixed for 5 min at room temperature. Jeffamine (50 μL, 1 M) was then added and the mixture was allowed to react for 3 h at room temperature. The Jeffamine-BSA conjugate was subsequently purified using a PD-10 column (GE Healthcare, Little Chalfont, UK). The freeze-dried Jeffamine-BSA (2 mg) was resuspended and both CYN (550 μg) and formaldehyde (12 μL) were added. The resultant mixture was allowed to react for 50 h, followed by dialysis over 24 h in 0.15 M saline solution.

### 3.3. Monoclonal Antibody Production

Hybridomas-producing anti-CYN monoclonal antibodies (MAbs) were prepared with a standard method for immunization and cell fusion. BALB/c mice were first immunized with 100 μg KLH-CYN in 0.1 mL sterile saline and 0.1 mL of complete Freund’s adjuvant. Then, the mice were boosted with KLH-CYN in incomplete Freund’s adjuvant with an internal of three weeks. When a high antibody titer was observed, a final immunization was performed intraperitoneally without adjuvant. The spleen cells of BALB/c mice immunized with KLH-CYN were fused with Sp2/0 cells. The resulting hybridomas were screened by indirect ELISA to select antibody-producing clones specifically reacting with BSA-CYN. Positive hybridomas were ultimately propagated in vitro, and then used for the preparation of ascites. This study has been approved and registered by the laboratory animal welfare and ethics committee of Southern Medical University (Guangzhou, China), and the care and use of the animals was conducted according to the Institutional Animal Ethics Committee guidelines.

Monoclonal antibodies were purified using a protein G column (ThermoFisher Scientific, Shanghai, China) according to manufacturer’s instructions. Verification of the purified antibody subclass was accomplished using a Pierce^®^ Rapid Isotyping Kit with Kappa and Lambda-Mouse from Thermo Scientific (Rockford, IL, USA).

### 3.4. Eu^3+^-Labeled BSA-CYN

To prepare the Eu^3+^-labeled BSA-CYN, 0.1 mg DTTA-Eu (N1-[P-isothiocyanato-benzyl]-diethylene-triamine-N1, N2, N3-tetraacetate-Eu^3+^) was added to 0.5 mg of BSA-CYN in 100 μL labeling buffer. The mixture was stirred and kept overnight at room temperature. The Eu^3+^-labeled BSA-CYN was then separated from unreacted chelates and aggregated proteins by Sephadex G-50 gel filtration using elution buffer. Fluorescence of the labeled BSA-CYN was measured at 615 nm and then aliquots of the labeled protein were kept at −20 °C.

### 3.5. Coating with Secondary Antibody

Goat anti-mouse IgG secondary antibody (200 μL) diluted to final concentration of 3 μg mL^−1^ with coating buffer was pipetted into each well, after which the plates were incubated at 4 °C overnight and then washed three times with washing buffer. Next, 250 μL of blocking buffer was added to each well and the samples were maintained at 4 °C overnight. Following removal of the blocking buffer, the plates were vacuum dried and stored with a desiccant at −20 °C.

### 3.6. Development of the Direct Competitive TRFIA

The assay was performed using the one-step procedure and the direct competitive protocol. Following immobilization and antibody labeling protocols described above, 100 μL of CYN standards (0, 0.01, 0.05, 0.1.0.5, 2, 5, 10, 20, 50 and 100 ng mL^−1^) or samples, 25 μL of assay buffer containing 400 ng N8 MAb, and 25 μL of assay buffer containing 20 ng of Eu^3+^-labeled BSA-CYN were added into each well and then coated with goat anti-mouse IgG antibody. A competition model in wells was generated subsequent to one hour of incubation with continuous slow shaking. The wells were then washed six times and filled with 100 μL of enhancement solution. The plates were then shaken for 5 min at room temperature and fluorescence intensity measured using a Victor3 1420 Multilabel Counter equipped with filters for Eu^3+^ (excitation wavelength, 340 nm; emission wavelength, 613 nm; delay time, 0.40 ms; window time, 0.40 ms; cycling time, 1.0 ms). Fluorescence intensities were corrected for Eu^3+^-labeled BSA-CYN binding without the presence of CYN by dividing the signal of the sample or standard solution (*F*) by that of the zero-concentration calibrator (*F*_0_). The logit-log method was used to generate a linear calibration curve.

### 3.7. Validation

#### 3.7.1. Analytical Sensitivity

Analytical sensitivity was determined by subtracting two standard deviations (SD) to the mean fluorescence value of 20 zero standard replicates and calculating the corresponding concentration.

#### 3.7.2. Precision and Accuracy of the Assay

To assess repeatability (intra-assay) and reproducibility (inter-assay), three CYN standards (2, 5 and 20 ng mL^−1^) were analyzed with the same batch of reagents on separate days.

#### 3.7.3. Spiked Sample Analysis

Negative water samples were filtered through Whatman GF/C filters, after which the filtrates were spiked with a 1 μg mL^−1^ CYN solution to achieve levels of 0.25, 5 and 25 ng mL^−1^. Each spiked sample was then analyzed in triplicate and the CYN recoveries were calculated by comparing measured and spiked values.

#### 3.7.4. Dilution Linearity Test

Serial dilutions of the CYN positive samples (10.0 and 50.0 ng mL^−1^) were made in assay buffer and the potency of each dilution was determined in triplicate using the developed TRFIA. Assay recovery was assessed by comparing observed and expected values.

### 3.8. Comparison of Sample Analysis with ELISA Kit

Water samples collected from the reservoirs in Guangdong province, South China, were first filtered through Whatman GF/C filters, with the filtrates subjected to CYN determination. The cultured *Cylindrospermopsis* samples were frozen at −20 °C; the cells were then lysed by freeze-thaw prior to measurement. Insoluble cell debris was removed by centrifugation for 10 min, and the supernatant was used for CYN detection. The samples were simultaneously analyzed using the TRFIA protocol as described above and a Beacon ELISA kit following manufacturer’s instructions.

### 3.9. Data Analysis

Statistical analysis of the data was performed using the Statistical Product and Service Solutions (SPSS) software (version 20.0, SPSS Inc., Chicago, IL, USA, 2011). A two-tailed test was applied for statistical analysis in all tests. A *p* value < 0.05 was considered statistically significant.

## 4. Conclusions

We successfully generated nine monoclonal antibodies against CYN, among which N8 had higher affinity to CYN than the other antibodies in relation to binding capacity. A direct competitive TRFIA based on this antibody was developed and validated. This is the first report employing this novel immunoassay to CYN detection. Along with the sensitivity and reliability, this makes the developed TRFIA an efficient tool for the monitoring of CYN in water and algal samples.

## Figures and Tables

**Table 1:** Precision and accuracy test of the newly developed TRFIA.

Type	Samples	Nominal Value (ng mL^−1^)	Mean ± SD (ng mL^−1^)	CV (%)	Recovery (%)
Intra-assay (*n* = 8)	A	2.0	1.91 ± 0.078	4.1	95.9
	B	5.0	5.16 ± 0.24	4.6	103.2
	C	20.0	20.3 ± 1.18	5.8	101.5
Inter-assay (*n* = 12)	A	2.0	1.96 ± 0.096	4.9	98.1
	B	5.0	5.08 ± 0.24	4.8	101.7
	C	20.0	19.5 ± 12.7	6.5	97.4

CV: coefficient of variation. SD: standard deviation.

**Table 2:** Recovery and coefficient of variation of CYN-spiked samples.

Spiked Value (ng mL^−1^)	Measured Value ± SD (ng mL^−1^)	Recovery (%)	CV (%)
0.25	0.246 ± 0.04	98.6	16.38
5.0	5.44 ± 0.54	108.8	9.99
25	23.15 ± 1.91	92.6	8.24

CV: coefficient of variation. SD: standard deviation.

**Table 3:** Dilution Linearity test for the newly developed TRFIA.

Sample	Dilution	Expected Value (ng mL^−1^)	Observed Value (ng mL^−1^, *n* = 3)	Recovery (%)
A	NA		10.0	
	1:2	5.0	4.86	97.2
	1:4	2.5	2.41	96.4
	1:8	1.25	1.29	103.2
	1:16	0.62	0.66	106.5
B	NA		50.0	
	1:2	25.0	25.4	101.6
	1:4	12.5	13.0	104.6
	1:8	6.25	5.91	94.5
	1:16	3.12	3.39	108.7

NA, not applicable.

**Table 4:** Comparison of assay performance for the newly developed TRFIA reagent and commercial ELISA kits.

Method	Recovery	Imprecision	Operating Time	Maximum Quantitative Value
TRFIA	95.9–103.2%	4.1–6.5%	1 h	50 ng mL^−1^
ELISA (Beacon)	80–120%	<20%	1.5 h	2 ng mL^−1^
ELISA (Abraxis)	98–108%	4.3–8.3%	1.25–1.5 h	2 ng mL^−1^
